# A Solitary Peutz-Jeghers Hamartomatous Polyp in the Gastric Body: A Case Report

**DOI:** 10.7759/cureus.63943

**Published:** 2024-07-06

**Authors:** Noelia Madera, Noemí Acevedo, Carmen González-Peralta, Rafael Castro, Vismelis Mezquita-Luna

**Affiliations:** 1 Internal Medicine, Clínica Corominas, Santiago, DOM; 2 Medicine, Pontificia Universidad Católica Madre y Maestra, Santiago, DOM; 3 Gastroenterology, Clínica Corominas, Santiago, DOM; 4 Pathology and Laboratory Medicine, Hospital Regional José María Cabral y Báez, Santiago, DOM

**Keywords:** histology, gastroenterology, hamartoma, peutz-jeghers syndrome, intestinal polyposis

## Abstract

Peutz-Jeghers syndrome (PJS) is a rare autosomal dominant genetic condition characterized by the development of hamartoma-type intestinal polyposis and areas of skin pigmentation, among other signs. Additionally, the occurrence of solitary Peutz-Jeghers polyps is exceedingly rare. We present the case of a 50-year-old female with a medical history of hypothyroidism, chronic gastritis, and dyslipidemia, who presented with dyspeptic symptoms and occasional rectal bleeding. Endoscopic examination revealed a solitary hamartomatous polyp in the gastric body and other gastrointestinal abnormalities. The patient underwent treatment and is being monitored with regular endoscopic studies and evaluations for other potential neoplasms. This case underscores the importance of considering the syndrome as a potential differential diagnosis. It emphasizes the necessity of a multidisciplinary approach to managing and monitoring such cases, particularly the early detection of possible neoplasms.

## Introduction

Hamartomatous polyps (HPs) are commonly associated with Peutz-Jeghers syndrome (PJS), characterized by gastrointestinal polyposis, mucocutaneous involvement, and a significant genetic component. If a patient has an HP but no mucocutaneous pigmentation or family history of PJS, they are diagnosed with a solitary Peutz-Jeghers polyp [1]. An incomplete clinical presentation of the phenotype is expected, making it essential to consider this pathology in differential diagnoses, mainly due to its association with neoplasms. This condition is linked to gastric metaplasia and shows a strong correlation in females with malignant neoplasms, such as breast cancer and malignant adenoma of the cervix [2]. The following case presents a patient with endoscopic and histopathological findings of a solitary HP.

## Case presentation

This case report details a 50-year-old female patient with a known medical history of hypothyroidism of unspecified duration, chronic gastritis with *Helicobacter pylori *(*H. pylori*), and dyslipidemia. Her conditions are managed with levothyroxine 50 mcg, amoxicillin 500 mg, rifabutin 150 mg, and rosuvastatin 20 mg, respectively. She presented to the gastroenterology clinic on an outpatient basis with constant heartburn, constipation, and occasional rectal bleeding.

Regarding previous diagnostic evaluations, she underwent a colonoscopy one year ago, which was poorly prepared and yielded inconclusive findings. Previous endoscopy results indicated findings consistent with a hiatal hernia, Barrett's esophagus, a gastric polyp in the fundus, and intestinal metaplasia.

**Figure 1:**
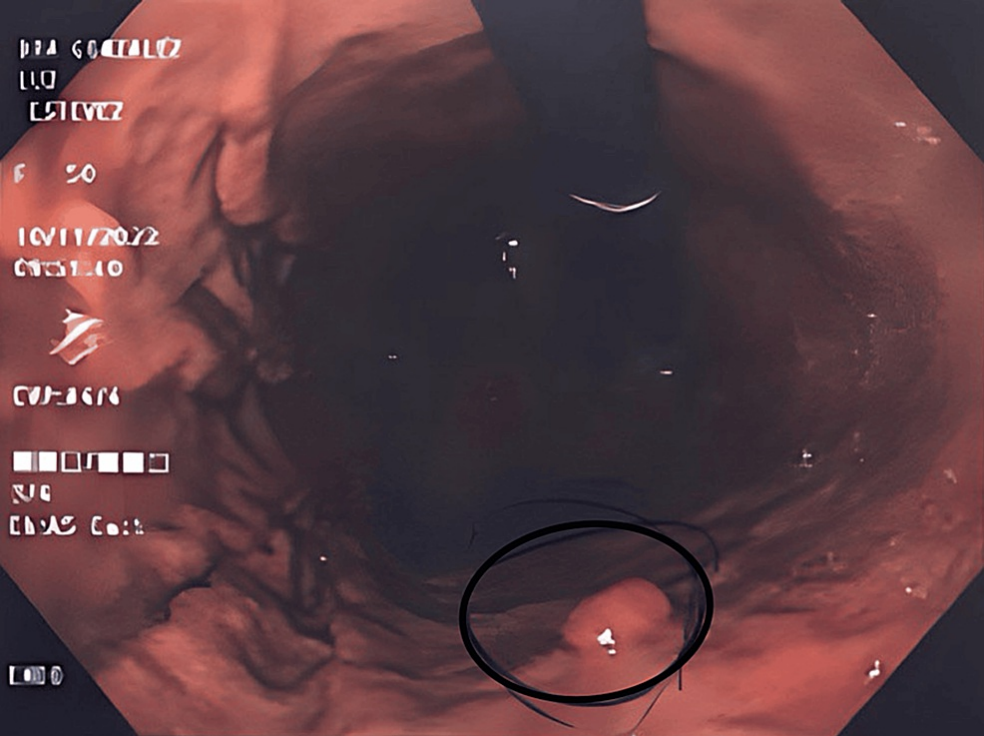
Elevated/polypoid lesion The circle shows a sessile polyp (0-IS), 12 mm in diameter in the body of the stomach

At the visit, it was recommended to initiate treatment with magnesium citrate to alleviate constipation, and endoscopic studies were ordered. Upper endoscopy revealed a hiatal hernia with no pathologies inside, universal erythematous gastropathy, and a 12 mm 0-IS polyp in the gastric body (Figure 1). Additionally, Forrest III pre-pyloric ulcers and infectious duodenitis were noted. During the procedure, a polypectomy with a diathermy loop was performed. Biopsy results showed moderate chronic gastritis activity and *H. pylori* positivity. The duodenal aspirate was positive for *Giardia lamblia*.

**Figure 2:**
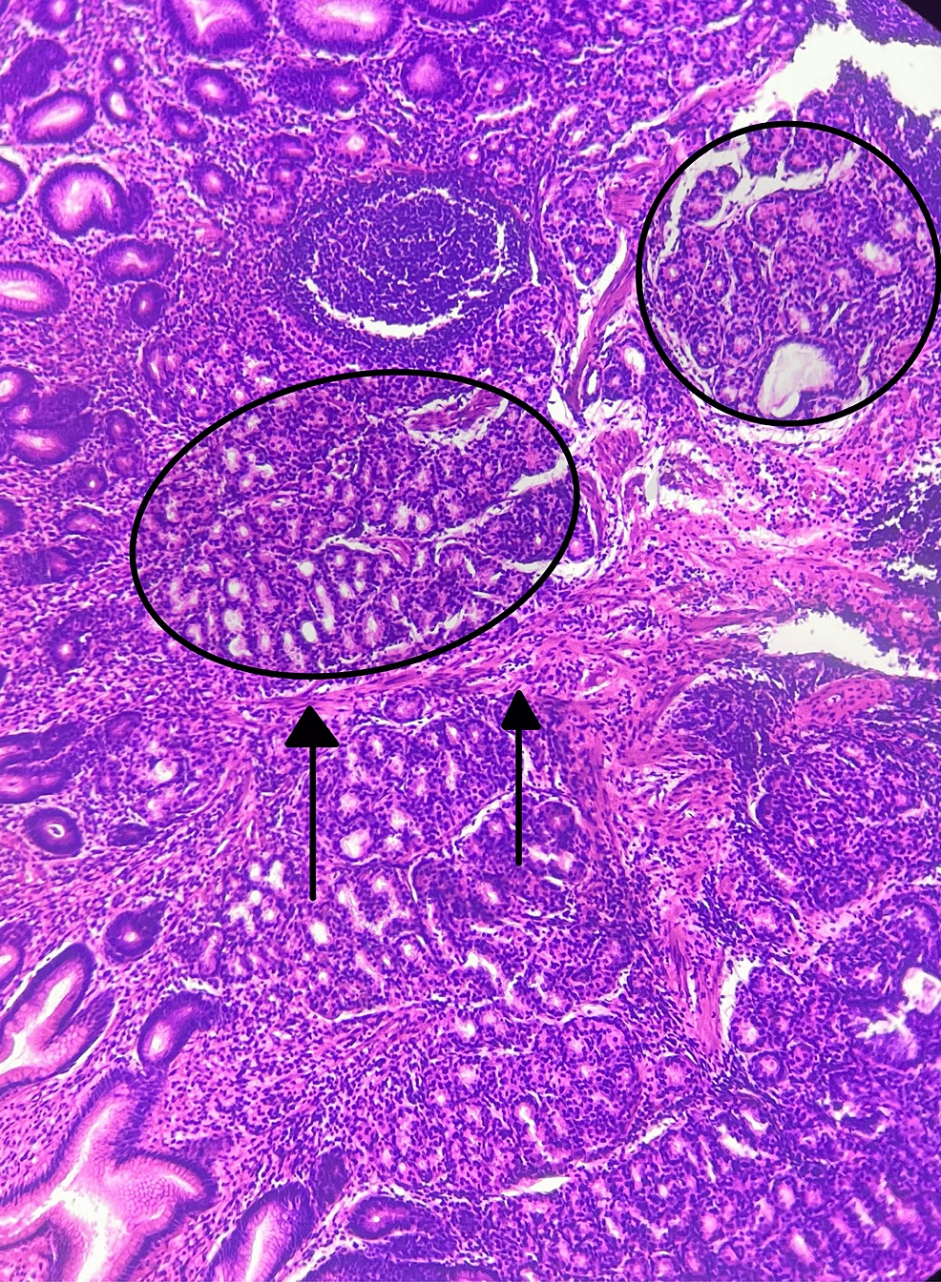
Hematoxylin and eosin (H&E) staining Circles show hyperplastic glands arranged in lobules, and arrows show smooth muscle fibers or septa among the arborizing glandular component

**Figure 3:**
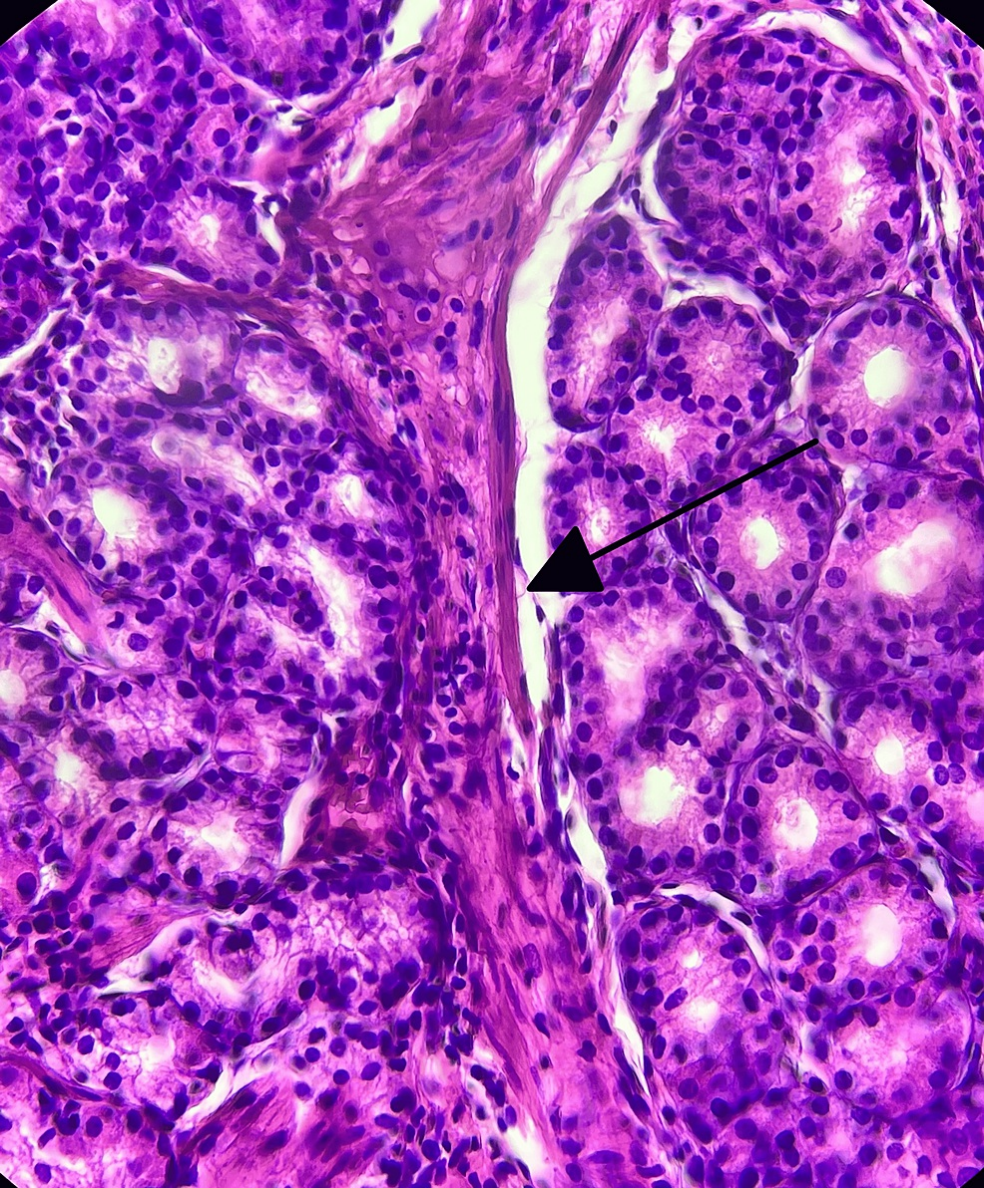
Hematoxylin and eosin (H&E) staining The arrow shows smooth muscle fibers among the arborizing glandular component

**Figure 4:**
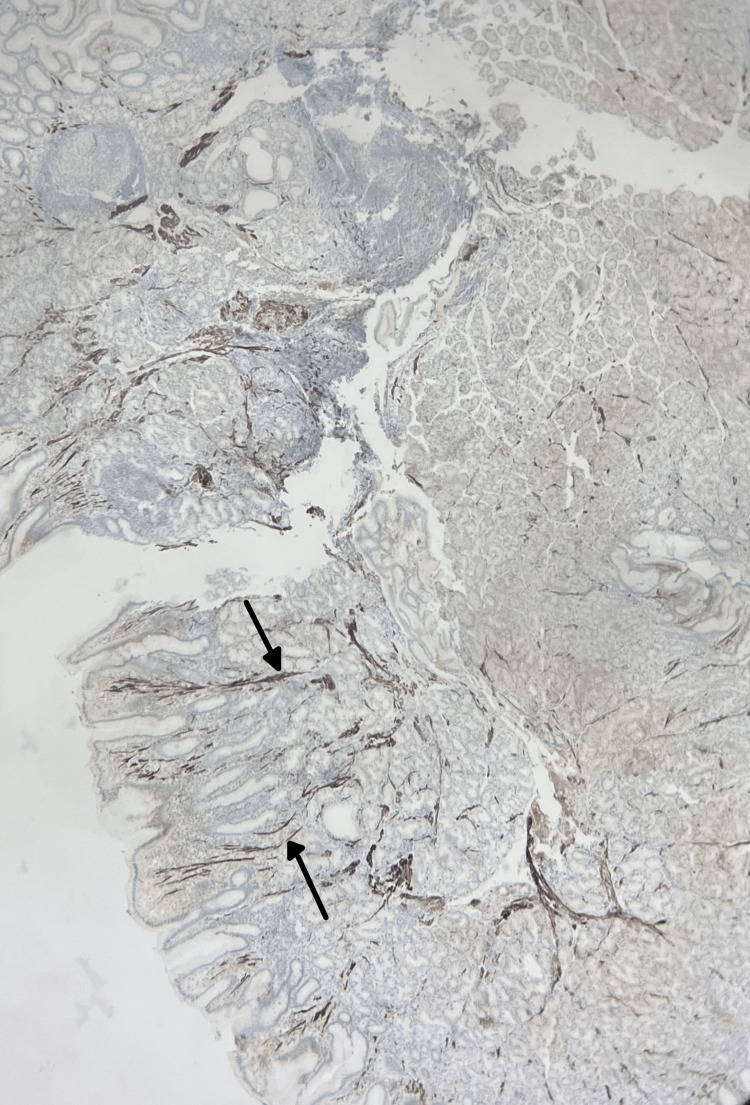
Immunohistochemistry (IHC) for desmin DE-R-11 compatible with hamartomatous polyp Arrows show smooth muscle fibers highlighted by immunohistochemistry for desmin

Lower gastrointestinal endoscopy scored 8 on the Boston scale and showed internal and external hemorrhoids. Biopsy results reported a moderately active polyp positive for HP, consistent with a Peutz-Jeghers gastric polyp without dysplasia (Figures 2-4). Capsule endoscopy showed no polyps in the path. Other laboratory studies showed no pathological findings.

Following this assessment, the patient was prescribed paromomycin 500 mg, esomeprazole 20 mg, clarithromycin 500 mg, amoxicillin 500 mg, and bismuth subsalicylate 300 mg. Given the increased risk of various epithelial malignancies, breast, thyroid, endometrial, and cervical health evaluations were indicated.

Follow-up mammography reported amorphous microcalcifications in the left breast. Subsequent breast biopsy reported adenosis without atypia, calcifications, usual hyperplasia, fibrosis, cysts, and elements of fibrocystic condition. Thyroid sonography indicated thyroiditis with no nodular lesions. Pelvic tomography and cervical cytology showed no pathological findings. An endometrial biopsy revealed mucoid and fibrohaematic material with few fragments of loose endometrial glands of atrophic appearance without atypia. Lastly, the endocervix biopsy showed the presence of squamous epithelium with reactive-looking nuclear atypia.

## Discussion

Gastrointestinal polyps are categorized into hyperplastic, inflammatory, adenomatous, and hamartomatous types [1]. The latter are extremely rare and occur mainly in PJS. The incidence of solitary Peutz-Jeghers polyps is extremely low [1,3]. These polyps can be found throughout the gastrointestinal tract, frequently in the small intestine and colorectal region, with the gastric location being the rarest [4]. HPs are characterized by the arborization of the smooth muscle bundle into the lamina propria with an almost normal overlying epithelium. Multiple polyps in the gastrointestinal tract, mucocutaneous symptoms, the STK11/LTB1 mutation, and family history are characteristics of PJS [5].

The clinical course of this pathology can present with gastrointestinal bleeding or obstructive symptoms [4]. In this case, the patient exhibited dyspeptic symptoms without other associated manifestations, prompting a more exhaustive investigation with digestive studies, which revealed a single polyp in the gastric body. The histopathological appearance of solitary polyps can be described as unique entities since they do not show the STK11/LTB1 mutation, and their occurrence in the gastric site is rare.

One limitation in this case was the lack of molecular genetic tests to confirm the diagnosis and monitor first-line relatives. The prognosis for these patients is generally good, with a low recurrence rate after polyp resection. However, malignant transformation and dysplasia cases have been observed in other parts of the body [6]. Given this, there is a recommendation, albeit with low-quality evidence, for periodic screening for primary neoplasm manifestations. There is still no consensus on the use of prophylactic mastectomy to reduce the risk of breast cancer, especially in those with other risk factors such as family history [6,7]. For this patient, an endoscopic digestive study was recommended every three years, in addition to an annual breast examination and genetic counseling.

## Conclusions

PJS represents a rare but significant condition characterized by gastrointestinal polyps and mucocutaneous pigmentation. While solitary gastric polyps are sporadic, their benign nature underscores the importance of accurate diagnosis and regular surveillance to detect potential complications such as malignancy. In developed countries, genetic testing for STK11/LTB1 mutations effectively guides management and surveillance protocols. However, in resource-limited settings, access to such diagnostics and specialized care remains challenging, highlighting healthcare delivery disparities. Increased awareness and collaborative efforts are essential to ensure timely diagnosis and appropriate management globally, aiming to mitigate risks and improve outcomes for individuals with PJS worldwide.
